# Effects of Temporary Numerical Imbalances on Collective Exploratory Behavior of Young and Professional Football Players

**DOI:** 10.3389/fpsyg.2019.01968

**Published:** 2019-08-27

**Authors:** Albert Canton, Carlota Torrents, Angel Ric, Bruno Gonçalves, Jaime Sampaio, Robert Hristovski

**Affiliations:** ^1^National Institute of Physical Education of Catalonia (INEFC), University of Lleida (UdL), Lleida, Spain; ^2^Research Centre in Sports Sciences, Health Sciences and Human Development, CIDESD, CreativeLab Research Community, Universidade de Trás-os-Montes e Alto Douro, Vila Real, Portugal; ^3^Faculty of Physical Education, Sport and Health, Ss. Cyril and Methodius University, Skopje, Macedonia

**Keywords:** tactical behavior, small-sided games, dynamic overlap, complex systems, state space

## Abstract

The aim of this study was to explore how the use of temporary numerical imbalances during small-sided Game SSGs affects team’s exploratory behaviors (i.e., variety and quantity of responses given in an ever-changing game context and its rate of change) in different age groups. Two different age groups (under−15 and under−23) of football players participated in the study. For each age group, three teams of five players played six small-sided games of 5 min duration in different conditions: (i) numerical balance (GK + 4 vs. 4 + GK); (ii) temporary numerical imbalance, which consisted of a numerical change of teammates and opponents every one minute. Latitude and longitude GPS coordinates were used to determine the positioning-derived variables. The dynamic overlap (i.e. the measure of average similarity of the game patterns that take place in increasingly larger time intervals) was used to provide information of the rate and breadth of exploratory behavior. The results revealed that the long-term exploratory breadth increased for the under−23 age group. Non-clear effects were found for the short-term rate of exploration, but with an increasing trend. In the under−15 group, the exploratory behavior was more likely to increase in the long term. The increase for the short-term rate of exploration was unclear, but it follows an increasing trend. These results suggest that the use of temporary numerical imbalances could offer coaches more dynamic training situations and different adaptive training environments similar to matches.

## Introduction

During a game of a team sport, different dynamic interpersonal coordination movements arise between the players and the environment. These team coordination movements can be captured from positioning-derived variables (i.e., from GPS/LPS or optical technology) that describe the coordinative patterns of a team in response to the set of game constraints (Ric et al., 2016). The most frequent team-related variables, resulting from the dynamic player’s positional data, used to reveal the tactical pattern of a football team and to define its tactical behavior and exploratory behavior are: player’s positioning in sectors and corridors of the field (Travassos et al., 2014), geometrical center of the team (Duarte et al., 2012), geometrical center of the team’s sectors (Gonçalves et al., 2013), team width and length (Frencken et al., 2011; Folgado et al., 2012) and spread rate, that is, the speed of change of the stretch index (STI) (Bourbousson et al., 2010). The STI is a tactical metric related to the spaces of play, which tries to understand the defensive principle of contraction (concentration of players) and the offensive principle of expansion (width and length), in the longitudinal and lateral axes that take place during a match or small-sided Game (SSG) due to the collective tactical behavior of the players of a team (Bourbousson et al., 2010; Clemente et al., 2013). These positioning-derived variables can be used to reveal the effect of relevant constraints on collective tactical behaviors during full and SSGs (Duarte et al., 2013; Gonçalves et al., 2016b).

A major aspect for the observation of game play is the configuration of play approach and its ever-changing shape, particularly via periods of contraction and expansion, and its dynamical position on the field (Gréhaigne and Godbout, 2014; Gonçalves et al., 2018). These game situations seem to be in symbiosis and one succeeds the other. It can help players to elucidate and anticipate the movement in the game and take right decisions (Gréhaigne and Godbout, 2014). In this sense, it seems reasonable to think that as found by Bourbousson et al. (2010) the STI of each team will present a bi-stable attraction in the longitudinal direction (while one team is in open space play in the opponent field the opponent team is in retreat close to their goal). The combination of two or more of these team variables would allow us to depict the state space of a team, where the high values define unstable coordinative states while the minimum values define highly stable coordinative states (Ric et al., 2017). For example, the STI and the distance of the centroid to the own goal (DCG) determine a pattern of interaction between two confronted teams (Gréhaigne and Godbout, 2014).

Exploratory behavior has been defined as a “subsequent realization of a large number of movement configurations which reveals the hierarchical action landscape under specific constraints of each performer” (Hristovski et al., 2011, p. 187) or team. Exploratory practice of unusual training environments may promote a varied and flexible behavior, so that performers learn to be more adaptive and, at the same time, more creative (Santos et al., 2018). Consequently, exploratory behavior seems to be important in collective sports because they are based on the improvisation and interaction between performers within the changing environment (Hristovski et al., 2012) and because it offers discovery of perception action solutions to the emergent tasks. There are some actions that are more likely to be repeated than others under the players/team and environmental interaction. In football, for example, this could be achieved by manipulating key tasks to direct the search of the apprentices so that they find effective coordination solutions (Davids et al., 2005), whether individual or collective. Thanks to the manipulation of tasks by the coach, the performers are encouraged to discover the answer to a task for themselves, without the need for the performer to receive instructions, orders or an exact list of those things that must be done (Hristovski et al., 2011).

In recent years, many studies have shown how the manipulation of constraints in SSGs can cause changes in individual and collective tactical behavior (Ometto et al., 2018). SSGs are specific format training tasks with the goal of reducing interactions and increasing the proportion of players who participate in the decision-making process, but keeping basic variability properties from the game (Aguiar et al., 2012; Davids et al., 2013). They are often adopted as a training drill used by coaches of different team sports, but the research into SSGs has focused particularly on football (Folgado et al., 2018; Coutinho et al., 2019), and to a lesser extent on basketball (Sansone et al., 2018), rugby (Vaz et al., 2019), futsal (Assunção et al., 2019) and handball (Iacono et al., 2015). These kind of games often simulate sub-phases of full-sided games preserving their unstable, dynamic and unpredictable nature (Hristovski et al., 2013; Gonçalves et al., 2016a; Ric et al., 2016) and they often reproduce the physical, technical and tactical (Bach Padilha et al., 2017) requirements of real match play (Aguiar et al., 2012). In order to replicate these demands in practice, various task constraints are manipulated by coaches, such as the pitch size and shape, the encouragement of the coach, the number of players involved in each team (Silva et al., 2014), the specific rules of the game, the duration of the game and rest periods, the available balls or the method by which players can score points (Hill-Haas et al., 2011). To describe the movements made by athletes during competition (Castellano and Casamichana, 2014) or to monitor different training tasks (Aughey, 2011), collective sports have applied the use of GPS technology (Castellano and Casamichana, 2014). Positional data has also allowed it to be demonstrated that during a football match, local and temporary numerical imbalances appear (Vilar et al., 2013). The numerical imbalances have been widely studied in recent years (Sampaio et al., 2014; Gonçalves et al., 2016b; Torrents et al., 2016). However, no research has evaluated the effects of the temporary manipulation of constraints during training. To train this kind of situation, coaches make use of numerical imbalances or joker players in SSGs (Ric et al., 2015), which are extra players placed internally or externally to the playing space, who can be fixed during the whole of the training drill or temporary. Their role is important insofar as it constrains the game by forcing the other players to adapt to the new game contexts (Ric et al., 2015).

To our knowledge, no research has evaluated the effects of temporary numerical imbalances on team exploratory behavior, as suggested by Ric et al. (2015, 2016). Thus, the aim of this study was to explore how the use of temporary numerical imbalances in SSGs affects the exploratory breadth of a team in different age categories.

## Materials and Methods

### Participants

The participants in the study were 30 male football players, of which 15 of them were under the age of 23 years old (age: 19.9 ± 1.6 years) and the other 15 were under the age of 15 years old (age: 13.8 ± 0.4 years). Each age groups were homogenous because they played in the same team and category (Under 23 group: Spanish 3rd division; under 15 group: División de Honor, top level of the Spanish football league system of that age). An informed and written consent was obtained from all adult participants and from the parents/legal guardians of all non-adult participants, before the beginning of the study. All participants were notified that they could withdraw from the study at any time. The investigation was approved by the local institutional Research Ethics Committee (CEIC Hospital Universitari Arnau de Vilanova) and it conformed to the recommendations of the Declaration of Helsinki.

### Procedure

For each age group, three teams (A, B and C) of five football players (four outfielders and a goalkeeper) played six SSGs games against each other (first, A vs. B; secondly A vs. C; and finally B vs. C) in two different SSG formats: balanced and imbalanced. Balanced SSG consisted of a fixed number of opponents (GK + 4 vs. 4 + GK) during the whole 5 min game. Imbalanced SSG consisted of numerical change of opponents and teammates every minute after the first minute, as follows: minute one: 4 vs. 4; minute two: 5 vs. 4; minute three: 4 vs. 5; minute four: 6 vs. 4; and minute five: 4 vs. 6. The three teams were distributed taking into account several factors according to the coach’s criteria, to ensure that the team’s performances were comparable (Aguiar et al., 2013). To ensure an equal distribution of players based on the team positions, teams and the changes of players were made under the coach criteria. All SSG were played on a natural pitch measuring 40 × 45 m, and in accordance with the official rules of soccer, with three exceptions to allow continuous spontaneous interactions between teammates and opponents (Davids et al., 2013; Torrents et al., 2016): first, there was no off-side; second, when a team scored, the same team kept the ball and restarted the game with a goal kick; third, when a ball was thrown out of the field limits, the game was restarted with a goal kick by the goalkeeper of the opposing team. These three actions were taken because they are usual in football training and to increase the effective playing time. In order to maintain the rhythm of play and avoid the influence of fatigue, each game involved 5-min periods of play separated by 3-min of passive rest (Table 1). The current study should acknowledge the limitation that no data related to fatigue was collected. To increase the effective playing time, the non-playing footballers and coaches were placed next to the goal to supply a ball whenever the game needed to be restarted.

**TABLE 1:** Data analysis for the SSG scenarios (Balanced and Imbalanced SSG) considering the variation in the number of opponents and teammates in the Temporary Numerical Imbalanced SSG.

**Time**	**Min 1**	**Min 2**	**Min 3**	**Min 4**	**Min 5**
Numerical balance	4 vs. 4	4 vs. 4	4 vs. 4	4 vs. 4	4 vs. 4
Temporary numerical imbalance	4 vs. 4	5 vs. 4	4 vs. 5	6 vs. 4	4 vs. 6

### Data Collection

Data were gathered through the use of a 5 Hz non-differential global positioning system (SPI ProX, GPSports, Canberra, ACT, Australia). The team-related variables used were: location of team geometrical centroid in the sectors and corridors of the field, the centroid speed, the team length and width, and the spread rate (Table 2). To define the field sectors and corridors we divided the field in six parts, from back to front and from right to left, respectively (Fradua et al., 2013). The team length is the distance between the most backward and the most forward player (*x*-coordinate) and the team width is the distance between the most lateral players on each side of the field (*y*-coordinate) (Frencken et al., 2011; Folgado et al., 2014; Frias and Duarte, 2015; Castellano et al., 2016). The spread rate is the velocity of contraction or expansion and was calculated by differentiating each STI data point of the time series to the following one. All these variables were determined using latitude and longitude coordinates exported from the GPS units and computed using dedicated routines in Matlab^®^ (MathWorks, Inc., MA, United States) (see the guidelines suggested by Folgado et al., 2014).

**TABLE 2:** Data collected to assess the tactical pattern of each team, formed by 36 categories.

**VARIABLE**	**CATEGORIES**
Sector (from right – 1 to left – 6)	0–7.5 m
	7.5–15 m
	15–22.5 m
	22.5–30 m
	30–37.5 m
	37.5–45 m
Corridor (from back – 1 to front – 6)	0–6.66 m
	6.66–13.33 m
	13.33–20 m
	20–26.66 m
	26.66–33.33 m
	33.33–40 m
Centroid Speed (m/s)	<−2
	−2 to −1
	−1 to 0
	0 to 1
	1 to 2
	>2
Team Length (m)	<4
	4 to 8
	8 to 12
	12 to 16
	16 to 20
	>20
Team Width (m)	<12
	12 to 16
	16 to 20
	20 to 24
	24 to 28
	>28
Speed of Spread Rate (m/s)	<−1
	−1 to −0.5
	0.5 to 0
	0 to 0.5
	0.5 to 1
	>1

A two-step cluster analysis was performed to determine the boundary values of each positioning-derived variable (Ric et al., 2016). The data collected for each team produced configuration states derived from the 36 categories belonging to the six variables named above. Teams changed their states during the 5-min game, so in total each game gathered 1500 vectors of data. Every vector was defined as a 36-component binary vector representing the full configuration state, attributing a value of 1 for active categories and 0 for the inactive ones. This enabled the formation of a 36 × 1500 multivariate binary (Boolean) matrix.

Two other variables, the STI and the distance from the centroid to the goal, allowed us to depict the potential landscape of team behavior. The STI was calculated by computing the mean distance of each team member from the spatial center for that team (Bourbousson et al., 2010; Frias and Duarte, 2015). The centroid is the geometric center of the average positions of the outfield players from a team (Frencken et al., 2011, 2012; Clemente et al., 2013; Folgado et al., 2014; Aguiar et al., 2015; Rein and Memmert, 2016). These two variables were used to capture and provide relevant information about collective organization of teams.

### Data Analysis

The dynamic overlap was computed to determine the dynamic properties of the game by defining these dynamics of either short or long-term exploration of player’s movement patterns (Ric et al., 2016). Therefore, it informs about the rate and breadth of the exploratory behavior during the game at different time-scales (Torrents et al., 2015). The dynamic overlap < *q_*d*_(t)* > was calculated as an average cosine auto-similarity between configurations for increasing time lag (Hristovski et al., 2013).

The mean dynamic overlap was fitted by the following equation, which is derived for systems with an intricate hierarchical structure (Sibani and Dall, 2003):

$$\left\langle q_{d}\,\left(t\right)\right\rangle =\left(1-q_{s\,t\,a\,t}\right)\,t^{-\text{α}}+q_{s\,t\,a\,t}$$

Where < *q_*d*_(t)* > is the known mean dynamic overlap, *q*_*stat*_ is the asymptotic value of the dynamic overlap (horizontal line of the curve which tends to infinity), *t* is the time lag, and α is the dynamic exponent (slope of the curve). *q*_*stat*_ detects the exploratory breadth of the team, and α the rate of exploration. Hence, the average dynamic overlap < *q_*d*_(t)* >, captures both the short- and long-term exploratory behavior of teams through the values of parameters α and *q*_*stat*_, respectively.

The combination of the distance from the team geometrical centroid to the own goal and the STI allowed us to define the state space of the teams for each condition and age group. Time series of both variables were divided in twenty clusters each allowing us to define a total of 400 configuration states. To determine each configuration state, the time series were clustered from the minimum distance from the centroid to the own goal (<8 m) to the maximum (>34 m) with a range of two meters, and one meter for the stretch index, from the minimum value (<4 m) to the maximum (>12 m). Probabilities of each configuration state were calculated by dividing the frequency of occurrence of each configuration (*n*_*i*_) by the total frequencies (*ρ_i_* = *n*_*i*_/*N*). The potential of each configuration state was calculated by the following equation (Balescu, 1975):

$$V_{i}=\mathrm{𝑄𝑙𝑛}\,\left(\rho_{i}\,/\,N\right)$$

Where Q is the standardized variance of the system assuming that noise is constant (*Q* = 1), and *N* is the total number of configurations.

### Statistical Analysis

Magnitude-based inferences and precision of estimation were applied to the inferential analysis due to the low sample (Batterham and Hopkins, 2006). Before the comparison of conditions (numerical balanced vs. temporary numerical imbalanced), all processed variables were log-transformed to reduce the bias from no uniformity of error. The variables used for the comparisons were the sector and corridor where the centroid was located (both from one to six), the speed of the centroid (m/s), the team length (m) and width (m), and the speed of spread rate (m/s). A descriptive analysis was performed using mean and standard deviations for each variable. Differences in means between scenarios were expressed in percentage units with 90% confidence limits (CL). The effect was reported as unclear if the CL overlapped the thresholds for smallest worthwhile changes, which were computed from the standardized units multiplied by 0.2. Magnitudes of clear effects were described according to the following scale: 25–75%, possibly; 75–95%, likely; 95–99%, very likely; > 99%, most likely (Hopkins et al., 2009). Also, the within-scenarios comparisons were assessed via standardized Cohen differences and respective 90% CL. Thresholds for effect sizes statistics were: 0.2, trivial; 0.6, small; 1.2, moderate; 2.0, large; > 2.0, very large (Hopkins et al., 2009).

## Results

Table 3 shows the results of α and *q*_*stat*_ values for numerical balanced and temporary numerical imbalanced conditions for each group of age and SSG condition. For the under−23 age group, the average value of *q*_*stat*_ most likely decreased (difference in means,%; ± 90% CL: −12.8; ± 2.7%) by using temporary numerical imbalances (0.24 ± 0.01). The effect size was very large (−2.32; ± 0.52) confirming the increase in the exploratory breadth. The rate of exploration (α) showed unclear effects for both situations. For the under−15 age group, the average value of *q*_*stat*_ unclearly decreased (−7.3; ± 14,8%) by using temporary numerical imbalances from a balanced situation. The rate of exploration (α) reported that its average value would be likely reduced (−6.4; ± 4.8%) from a numerical balanced situation to a temporary numerical imbalanced situation, with a small effect size (−0.35; ± 0.27).

**TABLE 3:** Results of *q*_*stat*_ and α values for numerical balanced and temporary numerical imbalanced conditions for each age group.

**Age group**	**Variable**	**Numerical Balance**	**Temporary Numerical Imbalance**	**Difference in means (%; ± 90%CL)**	**Chances for smaller/similar/greater**	**Uncertainty in the true differences**	**Standardized Cohen’s d (%; ± 90%CL)**
U23 (*n* = 6)	*q*_*stat*_	0.275 ± 0.013	0.239 ± 0.013	−12.8; ± 2.7	100/0/0	most likely ↓	−2.32; ± 0.52
	α	0.109 ± 0.011	0.106 ± 0.007	−2.3; ± 11.4	54/21/25	unclear	−0.22; ± 1.10
U15 (*n* = 6)	*q*_*stat*_	0.285 ± 0.048	0.264 ± 0.037	−7.3; ± 14.8	71/16/12	unclear	−0.45; ± 0.95
	α	0.109 ± 0.018	0.101 ± 0.013	−6.4; ± 4.8	88/11/1	likely ↓	−0.35; ± 0.27

*U, Under; CL, confidence limits; ↑, increase; ↓, decrease.*

**FIGURE 1:**
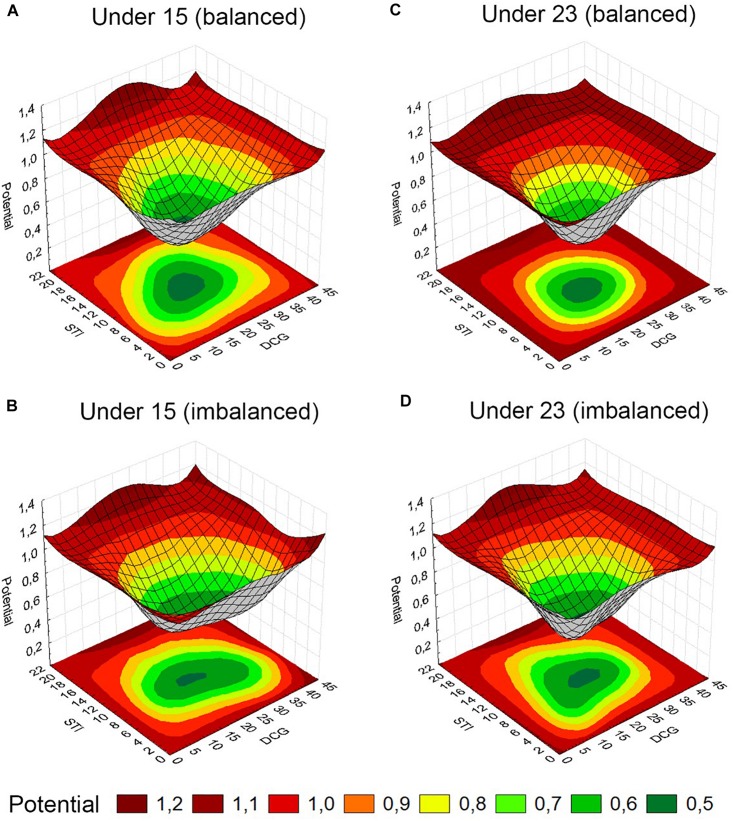
Attractiveness regions for team stretch index-distance from the centroid to the own goal under **(A)** under–15 years old balanced game, **(B)** under–15 years old in temporary numerical imbalanced game, **(C)** under–23 years old balanced game, **(D)** under–23 years old in temporary numerical imbalanced game. The 3D deeper wells correspond to 2D-projected more attractive (i.e., more probable) areas.

The relationship between the STI and the DCG is represented in Figure 1. The green shaded areas represent the attractive tactical behavior when relating STI and DCG, corresponding to the probability of their occurrence. The darker areas depicted in red are areas that can only be reached when the system is heavily destabilized. The pattern of a team will change depending on both the age of the players and the use of temporary numerical imbalances.

In accordance with these results, potential landscapes of both age categories showed that the use of extra players each minute create a wider basin of attraction allowing them to explore a greater number of tactical behaviors (Figure 1).

Related to a specific team behavior, these potential landscapes inform of the mid-field, which is closer to its own goal in the under−15 age group than in the under−23 age group. In other words, it seems that the under−23 players are able to keep their opponents further from their own goal. On the opponent’s mid-field, the same occurs in both age categories: the under−23 players cannot get as close to the opponent’s goal as the under−15 players.

## Discussion

This study shows that the manipulation of the number of teammates and opponents at 1-min intervals promoted, in the under−15 years of age category, a slight increase in the exploratory behavior in both short- and long-term exploration breadth; while in the under−23 years of age category, the same constraint promoted an unclear increase in the short-term exploration, and a very large increase in the long-term.

By using extra players that enter and leave a game over 60 s, new environments (training tasks) were created over shorter timescales allowing teams to quickly explore all their available state space (i.e., whole set of possible configuration of play). In that sense, it has been demonstrated that teams are able to adapt their behavior to perturbations of the environment (McGarry et al., 2002). Specifically, to real changing-game constraints in terms of local (interaction of few players) and temporary (manipulation of numerical task conditions on a shorter timescale) constraints (Vilar et al., 2013). Although in the present study the results did not show clear effects for the under−15 age group, professional teams increased their collective exploration.

According to these results, and in line with the suggestions of Ric et al. (2016), the use of temporary numerical imbalances in SSG seems to be justified if the objective of the coach is to promote a quick exploration of the whole variety of team behaviors. However, one must keep in mind that the age of the players can affect the exploration of the whole possibilities of action of the team (Almeida et al., 2016). Torrents et al. (2016) suggested that the easier a game situation is, the more regular and less varied play is promoted, and vice versa, the more difficult a scenario is, the more possible it is for the players to explore tactical actions to perform until a limit (too difficult scenarios would also promote a regular and less varied play). In line with this comment, we can consider that the temporary numerical imbalance situation carried out in the present study is a more difficult scenario for the players than a stable one, in terms of perception of the environment and when relating with varying numbers of teammates and opponents. It would explain why the exploratory behaviors are enhanced. In line with this, Vilar et al. (2014) concluded in a study where they examined the effects of the variation of the number of players involved in football SSGs on different individual actions, that these variabilities in the number of teammates and opponents are learning environments that will allow players a major transferability from one situation (training) to another (real match), and help them to perceive better the information sources and perform better according to their capabilities. Furthermore, these imbalances may promote different collective actions such as the reduction of the team area of play when in inferiority or less commitment in maintaining a pre-structured strategical behavior when playing in superiority conditions (Sampaio et al., 2014), offering the coaches an applicable approach to regulate the players perception under certain conditions (Vilar et al., 2014).

Taking into account the results of the potential landscapes, in which seems to exist a pressure exerted by the teams in order not to allow the opponent to get close to their own goal, we generated an hypothesis from the results of Bourbousson et al. (2010) which consisted of finding two main configurations of play, one of these configurations consisted of a compact team when located in its own mid-field, while the other configuration consisted of an expanded team in the opponent’s mid-field. Although our results do not concur with the Bourbousson et al. (2010) results, they are closer to the Moura et al. (2016) findings, which detected that when one team expands, the other team also does so, and vice versa.

Therefore, the use of this type of task constraint implies on the one hand changing the patterns of tactical behavior and the exploratory dynamics of the players by experiencing dynamic training situations that simulate the intrinsic variability of the competitive environment (Pinder et al., 2012; Davids et al., 2013; Ric et al., 2015; Torrents et al., 2015) and, therefore, allow the player to better solve those imbalanced situations that locally take place during a real game (Vilar et al., 2013). On the other hand, it reveals the differentiated decision-making abilities that performers are constrained to develop under varying conditions (Ric et al., 2016).

It would be interesting to carry out future research that continues to investigate the use of temporary numerical imbalances during SSGs to see how they affect the exploratory behavior of athletes in general and football players in particular and, if possible, comparing different age groups in order to help as many coaches as possible to create new training tasks that help them improve both the performance of their teams and their players. A concrete proposal is to perform different balanced and dynamic imbalanced training tasks in different age categories, but varying not only the number of players but also the field size to see how it modifies the tactical structure and the exploratory behavior of a team with the aim of elucidating which tasks are more prone to favor these two given variables. By creating tasks and generating different sub-tasks with different time constraints, these sub-tasks could be compared in different age categories to verify when the stationary state is achieved for each group of age. This would help coaches to choose more suitable tasks to favor the exploratory behavior of a team. Another future proposal is to compare how these time, size or numerical imbalance constraints affect different teams with different skill levels.

## Conclusion

The use of temporary numerical imbalances at 1-min intervals promotes the exploratory behavior of football players. However, it occurs differently depending on the age of the players. Accordingly, higher values were found in the short-term exploratory breadth in the under−15 years of age category, while in the long-term exploratory breadth happened the opposite. Considering that imbalances appear in different zones of the field at different moments, we suggest to create and implement such training tasks for its representativeness of real game. Moreover, taking into account that it is a task that both, promotes the exploratory behavior and it’s representative of what happens in a real match, it would be interesting to carry out this kind of activity in a learning context to guide learners to experience more game contexts that can later happen in the game. Thus, the findings of this study suggest that the use of temporary numerical imbalances will be useful for football players to perform more varied situations similar to real game. In this sense, we propose to perform future research in which other age categories and inter-team number imbalances are taken into account. Additionally, it would be of interest to know as to whether throughout the 1-min time epochs, there were varying expressions of the six variables investigated at different time periods (i.e., did a period of adjustment exist as players re-familiarized with having a numerical advantage or disadvantage?) by considering the variables as time series.

## Data Availability

The datasets generated for this study are available on request to the corresponding author.

## Ethics Statement

All participants were informed about the research procedures; players under the age of 23 years old provided prior informed consent, while for the players under the age of 15 the prior informed consent was provided by a parent or a legally authorized representative. The local Institutional Research Ethics Committee approved the study, which also conformed to the recommendations of the Declaration of Helsinki.

## Author Contributions

AC worked on the design of the study, collection, analysis, and interpretation of the data, and drafting of the manuscript. CT participated in the conceptualization and design of the study, and reviewed the content of the manuscript. AR participated in the conceptualization and design of the study, data collection, and reviewed the content of the manuscript. BG worked on the data collection and reviewed the content of the manuscript. JS reviewed the content of the manuscript. RH conceived the approach to data analysis, data interpretation, and drafting of the manuscript. All authors approved the final version of the manuscript and agreed to be accountable for all aspects of the work.

## Conflict of Interest Statement

The authors declare that the research was conducted in the absence of any commercial or financial relationships that could be construed as a potential conflict of interest.
